# Delivering an mRNA vaccine using a lymphatic drug delivery device improves humoral and cellular immunity against SARS-CoV-2

**DOI:** 10.1093/jmcb/mjac041

**Published:** 2022-07-08

**Authors:** Runqiang Chen, Hui Xie, Sahba Khorsandzadeh, Madison Smith, Namir Shaabani, Qidong Hu, Xiaoxuan Lyu, Hua Wang, Wan-lin Lim, Haotian Sun, Henry Ji, Brian Cooley, Russell Ross, David M Francis

**Affiliations:** Sorrento Therapeutics, Inc., San Diego, CA 92121, USA; Sorrento Therapeutics, Inc., San Diego, CA 92121, USA; Sorrento Therapeutics, Inc., San Diego, CA 92121, USA; Sofusa, a Division of Sorrento Therapeutics, Atlanta, GA 30350, USA; Sorrento Therapeutics, Inc., San Diego, CA 92121, USA; Sofusa, a Division of Sorrento Therapeutics, Atlanta, GA 30350, USA; Sorrento Therapeutics, Inc., San Diego, CA 92121, USA; Sorrento Therapeutics, Inc., San Diego, CA 92121, USA; Sorrento Therapeutics, Inc., San Diego, CA 92121, USA; Sorrento Therapeutics, Inc., San Diego, CA 92121, USA; Sorrento Therapeutics, Inc., San Diego, CA 92121, USA; Sorrento Therapeutics, Inc., San Diego, CA 92121, USA; Sorrento Therapeutics, Inc., San Diego, CA 92121, USA; Sorrento Therapeutics, Inc., San Diego, CA 92121, USA; Sofusa, a Division of Sorrento Therapeutics, Atlanta, GA 30350, USA; Sorrento Therapeutics, Inc., San Diego, CA 92121, USA; Sofusa, a Division of Sorrento Therapeutics, Atlanta, GA 30350, USA; Sorrento Therapeutics, Inc., San Diego, CA 92121, USA; Sofusa, a Division of Sorrento Therapeutics, Atlanta, GA 30350, USA

**Keywords:** COVID-19, lymph nodes, lymphatics, vaccines, drug delivery

## Abstract

The exploration and identification of safe and effective vaccines for the SARS-CoV-2 pandemic have captured the world's attention and remains an ongoing issue due to concerns of balancing protection against emerging variants of concern while also generating long-lasting immunity. Here, we report the synthesis of a novel messenger ribonucleic acid encoding the spike protein in a lipid nanoparticle formulation (STI-7264) that generates robust humoral and cellular immunity following immunization of C57Bl6 mice. In an effort to improve immunity, a clinically focused lymphatic drug delivery device (MuVaxx) was engineered to modulate immune cells at the injection site (epidermis and dermis) and draining lymph node (LN) and tested to measure adaptive immunity. Using MuVaxx, immune responses were elicited and maintained at a 10-fold dose reduction compared to traditional intramuscular (IM) administration as measured by anti-spike antibodies, cytokine-producing CD8 T cells, neutralizing antibodies against the Washington (wild type) strain and South African (Beta) variants, and LN-resident spike-specific memory B cells. Remarkably, a 4-fold-elevated T cell response was observed in MuVaxx-administered vaccination compared to that of IM-administered vaccination. Thus, these data support further investigation into STI-7264 and lymphatic-mediated delivery using MuVaxx for SARS-CoV-2 and VoC vaccines.

## Introduction

The severe acute respiratory syndrome coronavirus 2 (SARS-CoV-2) virus has accounted for >210 million cases of the coronavirus disease 2019 (COVID-19) and >4.4 million fatalities worldwide since its original outbreak in December 2019. This is the third outbreak of a betacoronavirus since 2002, the SARS-CoV and Middle East respiratory syndrome coronavirus being its predecessors, and it is much more efficiently transmitted from person to person. While vaccines have greatly blunted the spread of disease, questions about their durability and protection against emerging variants of concern (VoCs) remain (Pegu et al., 2021; Puranik et al., 2021). Furthermore, the origin of emerging VoCs is often in places that lack vaccine access equality; thus, vaccine development needs to be adapted to the regional needs of developing nations, including supply chain issues and healthcare worker training (Arya and Prausnitz, 2016). This is in addition to the urgent need to improve vaccine durability and efficacy to VoCs, while balancing costs, stability, and manufacturing speed to scale up worldwide vaccination efforts.

There are currently two messenger ribonucleic acid (mRNA)-based SARS-CoV-2 vaccines that have been authorized and widely disseminated. These vaccines encode the spike (S) protein, which is the major surface protein on the coronavirus virion, as it facilitates viral entry into host cells via interactions with the angiotensin-converting enzyme 2 (ACE2) receptor expressed in the upper and lower respiratory tract (Lukassen et al., 2020). Specifically, the S protein is composed of two subunits, S1 and S2, with the receptor-binding domain (RBD) located in the S1 subunit, which is responsible for directly binding to ACE2 (Liu et al., 2020; Wrapp et al., 2020). An effective vaccine should elicit antibodies against the S protein, including neutralizing antibodies (nAbs) specific to RBD to prevent viral entry into cells, and against S1 for broader protection, as it is exposed on the virion surface compared to the S2 subunit. In addition to humoral immunity, an ideal vaccine would also generate cellular immunity, as T cell immunity has been associated with less severe disease (Sekine et al., 2020; Tan et al., 2021), faster recovery (Tan et al., 2021), and memory persistence for decades (Le Bert et al., 2020).

To date, most vaccines are administered intramuscularly (IM) due to feasibility for healthcare workers, speed of injection, and immunological properties, i.e. muscle-resident lymphocytes and antigen-presenting cells (APCs). However, directing vaccines to lymph nodes (LNs) provides a promising opportunity for improving vaccine efficacy, as (i) lymphocytes reside in LNs at high concentrations (Schudel et al., 2019), (ii) LNs are home to unique and strategically positioned APCs that present incoming antigen to T and B cells rapidly to induce immunity (Pape et al., 2007; Gerner et al., 2015), and (iii) memory T and B cells reside in LNs during their lifespans (Woodland and Kohlmeier, 2009; Moran et al., 2018). Injection of vaccines into the epidermis and dermis may improve LN delivery as the initial lymphatics reside at high concentrations just below the stratum corneum and provide direct access to draining LNs due to their high permeability and uni-directional flow toward draining LNs (Swartz, 2001; Pal and Ramsey, 2011; Trevaskis et al., 2015). Moreover, high concentrations of APCs, including Langerhan cells, reside in the epidermis and dermis, which are highly efficient at taking up antigens and subsequently trafficking to draining LNs to elicit adaptive immunity (Romani et al., 2010; Kim et al., 2011; Thomas et al., 2012; Francis et al., 2020). Thus, superficial administration of vaccines into the epidermis and dermis presents a promising approach for enhancing vaccine efficacy.

Intradermal injections for vaccines have been of interest for decades (Hickling et al., 2011). However, they require a skilled healthcare worker to successfully administer the drug in the dermal layer without injecting them into the subcutaneous layer, which may limit large-scale use. To overcome this challenge and enable delivery to draining LNs, we engineered Sofusa MuVaxx (MuVaxx), a proprietary lymphatic drug delivery device. MuVaxx is derived from our core technology consisting of microneedles draped with a heat-formed nanotopographical imprinted polymer film over each microneedle with a through-channel to allow delivery, thereby increasing drug permeability through the skin (Walsh et al., 2015; Aldrich et al., 2017; Kwon et al., 2019). MuVaxx is an attachable device to any leur lock syringe and consists of 16 microneedles (4 × 4 pattern) with a height that delivers drugs to the epidermal–dermal junction where the initial, highly permeable lymphatic capillaries vascularize the skin tissue, resulting in improved lymphatic uptake (Skobe and Detmar, 2000). Moreover, we have previously demonstrated that the microneedles do not reach the pain receptors in the skin, leading to near pain-free injections (Kwon et al., 2019). MuVaxx does not require any drug modifications other than the drug that must be solubilized in a liquid; thus, it can be used with a variety of different drug modalities, including protein- and mRNA-based modalities for vaccinations.

To address the SARS-CoV-2 pandemic and the need for effective vaccines, we synthesized a novel mRNA construct encoding the S protein in a lipid nanoparticle (LNP) formulation—referred to as STI-7264—and interrogated the immunological response using a clinically standard IM injection compared to an intra-epidermal/dermal injection using MuVaxx. We first explored the ability of MuVaxx to deliver antigens to LNs to induce an improved immunological response by vaccinating against a model antigen, ovalbumin (OVA), in a preclinical mouse model compared to an IM injection. We next compared STI-7264 against a marketed reference mRNA-LNP (reference) vaccine for comparison and characterized the humoral and cellular response in a preclinical mouse model measuring circulating anti-S antibodies and peripheral cytokine-producing T cells. Our STI-7264 formulation led to similar antibody production compared to the reference vaccine; however, the CD8 T cell response was dramatically improved. MuVaxx administration of STI-7264 enabled a ∼10-fold reduction in the dose needed compared to an IM injection while maintaining similar B cell immunity as measured by anti-S1 and anti-RBD IgG. Moreover, MuVaxx elicited increased numbers of circulating cytotoxic CD8 T cells and memory B cells within draining LNs, highlighting its potential for cellular immunity and memory protection. Thus, the combination of STI-7264 and MuVaxx represents an innovative approach for vaccinating against SARS-CoV-2, and its VoCs as well as demonstrating results that move forward to clinical applications.

## Results

### STI-7264-SARS-CoV-2 mRNA is an mRNA vaccine for the prevention of COVID-19

**Figure 1:**
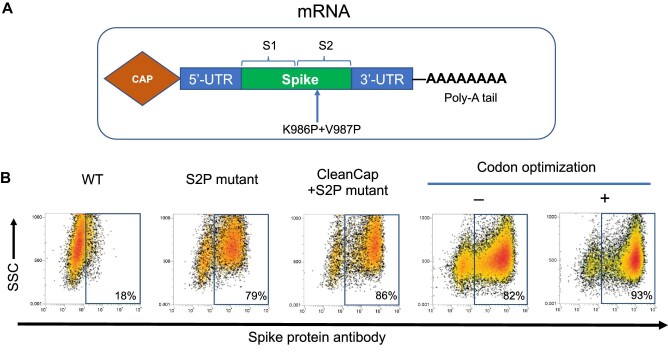
STI-7264 mRNA vaccine is optimized for highly efficient translation. (**A**) Schematic for STI-7264 mRNA sequence. (**B**) Primary DCs were transfected with various mRNAs, stained with the anti-S antibody STI-2020, and evaluated by flow cytometry at 24 h post-transfection.

The vaccine is comprised of an active drug substance and a single-stranded mRNA encoding the full-length SARS-CoV-2 S glycoprotein, which is encapsulated in LNP formulations specifically suited for the delivery of mRNA encoding the S protein. The sequence was derived from the strain ‘SARS-CoV-2 isolate Wuhan-Hu-1’. Mutations were introduced into the S protein to substitute residues 986 and 987 to produce a prefusion-stabilized SARS-CoV-2 S(2P) protein (Wrapp et al., 2020). To achieve optimal expression in humans, the sequence was further codon-optimized and cloned into a pVAX1-based backbone that contains T7 promoter, 5′-Untranslated region (UTR), 3′-UTR, and an optimized Poly-A tail with minimal overhang. The template was then linearized immediately downstream of the Poly-A tail and used for *in vitro* transcription (IVT) (Figure 1A). To facilitate mRNA expression and reduce innate immune response, during the IVT, Cap 1 structure was added to the 5′ terminus of the RNA co-transcriptionally by CleanCap^®^ AG, and uridine triphosphate (UTP) was completely replaced by N1-methylpseudo-UTP. This process can be readily scaled up to produce desired amounts of capped mRNA.

To confirm the expression, the IVT mRNA was introduced into monocyte-derived dendritic cells (DCs) by electroporation. Twenty-four hours post-transfection, the cells were collected and stained with anti-S antibody STI-2020 and detected with allophycocyanin-conjugated anti-human Fc antibody. Flow cytometry showed that codon-optimized Cap 1 mRNA can be efficiently translated into prefusion-stabilized S protein in primary DCs (Figure 1B).

### MuVaxx delivers antigen to LNs and improves immunity against a protein antigen

**Figure 2:**
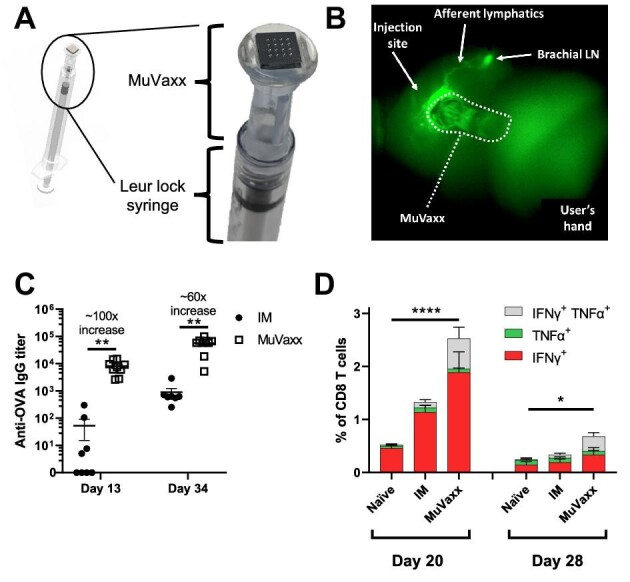
MuVaxx enables delivery to draining LNs and improves immunity compared to IM administration. (**A**) Schematic of the MuVaxx device connected to a 1 ml syringe. (**B**) Image of a C57Bl6 mouse at 5 min after injection of ICG using MuVaxx. (**C** and **D**) C57Bl6 mice were vaccinated with 10 μg OVA and 8 μg CpG on Days 0 and 14. (**C**) Serum was collected on Days 13 and 34, and anti-OVA IgG was quantified via serial dilutions run by ELISA assays. (**D**) Whole blood was collected and stimulated with SIINFEKL peptide followed by ICS to measure IFNγ and TNFα in the CD8 T cell compartment. Data in **C** and **D** represent two independent experiments (*n* = 18–19 mice per group). Cytokine statistics represent the difference between IFNγ^+^ groups for panel **D**.

To explore the potential of delivering vaccine constituents to draining LNs, we next utilized MuVaxx (Figure 2A) to deliver the drug at the epidermal/dermal boundary. To evaluate LN delivery using MuVaxx, we first used Indocyanine Green (ICG), which can be visualized *in vivo* with near-infrared fluorescence (NIRF). Following injection, we observed ICG accumulation within minutes in the draining brachial LN (Figure 2B). We then explored the potential of MuVaxx to augment the immunogenicity of a model antigen, specifically OVA in mice. Mice were injected with OVA and an oligonucleotide adjuvant (CpG) on Days 0 and 14 using an IM or MuVaxx administration. All mice treated with MuVaxx generated anti-OVA IgG by Day 13, compared to 4 of 8 in the IM cohort. Additionally, following the booster shot (Day 14), anti-OVA IgG was measured on Day 34, and mice treated with MuVaxx administration displayed significantly higher titers compared to mice treated with IM administration, resulting in a>60-fold increase in titers (Figure 2C). The cellular immune response was additionally measured in both cohorts of mice, looking at cytokine production in CD8 T cells following the booster shot on Days 20 and 28. Interferon-gamma (IFNγ) and tumor necrosis factor-alpha (TNFα) were assessed following *ex vivo* stimulation with SIINFEKL, an OVA-derived class I peptide. Mice treated with MuVaxx administration displayed higher proportions of cytokine-producing CD8 T cells compared to naïve mice on both Days 20 and 28 (Figure 2D). Taken together, these results highlight the potential of MuVaxx to deliver cargo to draining LNs to improve the immunity to vaccines.

### STI-7264 induces anti-S antibodies with MuVaxx enabling dose sparing

**Figure 3:**
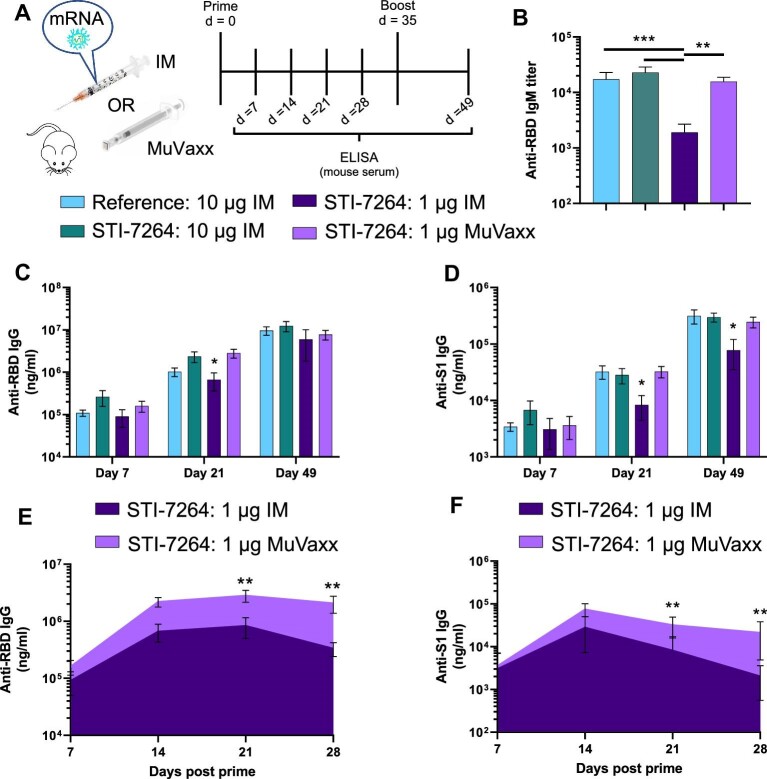
MuVaxx improves humoral immunity of spike-encoding mRNA-LNP formulations. (**A**) Treatment schedule. (**B**–**F**) Serum was collected and tested for spike-specific antibodies. (**B**) Anti-RBD IgM on Day 7. (**C**) Anti-RBD IgG on Days 7, 21, and 49. (**D**) Anti-S1 IgG on Days 7, 21, and 49. (**E**) Anti-RBD IgG AUC plots comparing IM and MuVaxx at 1 μg dose. (**F**) Anti-S1 IgG AUC plots comparing IM and MuVaxx at 1 μg dose. Data represent one experiment (*n* = 5 mice per group). Statistics in **C** and **D** represent significance against 1 μg STI-7264 IM vs. all other groups on that day.

To evaluate the potential of MuVaxx to enhance immunity, we compared the humoral immunity of STI-7264 against the reference vaccine along with the comparison of IM vs. MuVaxx administrations. C57Bl6 mice were immunized with either 10 or 1 μg of mRNA, specifically 10 μg reference IM or STI-7264 at 10 μg IM, 1 μg IM, or 1 μg MuVaxx, on Days 0 and 35 with serum collection every 7 days (Figure 3A). Seven days after the original primer shot, both 10 μg mRNA-LNP formulations (reference and STI-7264) when administered IM showed high anti-RBD-specific IgM responses, with a reduction in IgM observed for the 1 μg dose. Interestingly, when 1 μg STI-7264 was administered using MuVaxx, a similar IgM response was measured (Figure 3B). Comparable trends were observed when quantifying the IgG response for both anti-RBD and anti-S1-specific IgG antibodies (Figure 3C and D), with similar trends observed when administering 0.01 μg of mRNA via MuVaxx (Supplementary Figure S1), highlighting the dose-sparing potential that may be induced with MuVaxx. Additionally, when exploring the IgG response following the initial dose, MuVaxx administration elicited sustained anti-S IgG antibodies in the serum compared to the traditional IM administration that waned at a faster rate (Figure 3E and F). Overall, these results highlight the successful generation of a spike-encoding mRNA vaccine with delivery toward draining LNs and/or cells within the epidermis via MuVaxx, enabling dose-sparing activity.

### STI-7264 favors Th1 response over Th2

**Figure 4:**
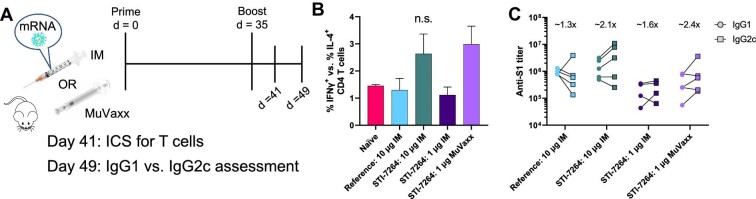
STI-7264 and MuVaxx bias response toward Th1 population. (**A**) Treatment schedule. (**B**) Whole blood was incubated with spike-associated peptides overnight followed by ICS. The ratio of CD4 Th1 (CD4 IFNγ^+^) to Th2 (CD4 IL-4^+^) phenotypes from whole blood on Day 41 is shown. (**C**) Serum was assessed by ELISA for S1-specific IgG1 and IgG2c on Day 49 by end-point titers. Numbers listed above each group represent average IgG2c:IgG1 ratio. Data represent one experiment (*n* = 5 mice per group).

To assess the Th1/Th2 bias elicited after immunization, whole blood was collected to measure cytokine-producing CD4 T cells 6 days after the booster shot via intracellular cytokine staining (ICS) and IgG subclass titers from the Day 49 serum (Figure 4A). The ratio of Th1 (CD3^+^CD4^+^IFNγ^+^) to Th2 (CD3^+^CD4^+^IL4^+^) T cells favored a Th1 response and was similar among all cohorts, although the 10 μg STI-7264 IM and 1 μg STI-7264 MuVaxx groups had slightly higher ratios, favoring an enhanced anti-viral immune response (Figure 4B). Similarly, the ratio of IgG2c to IgG1 was skewed toward IgG2c for the 10 μg STI-7264 IM and 1 μg STI-7264 MuVaxx cohorts, suggesting bias toward a Th1 response (Figure 4C) in line with the CD4 T cell cytokine phenotypes.

### Elevated CD8 T cell immunity is elicited following vaccination with STI-7264 and MuVaxx

**Figure 5:**
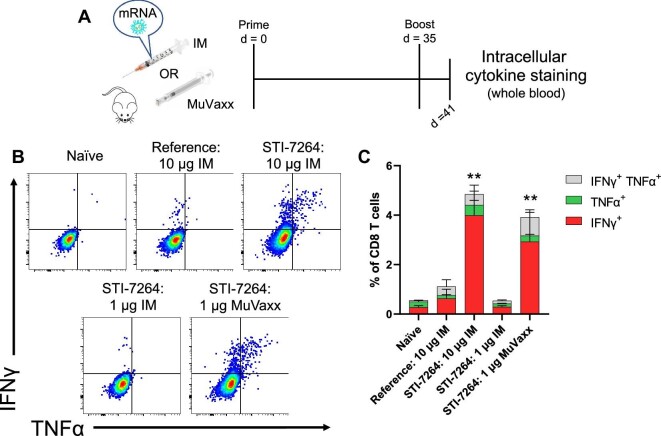
STI-7264 formulation improves CD8 T cell immunity. (**A**) Vaccine experiment timeline: 6 days following booster shot, ICS was performed in the presence of spike-associated peptides. (**B**) Representative flow cytometry plots of IFNγ and TNFα production from CD8 T cells. (**C**) Quantification of **B**. Data represent one experiment (*n* = 5 mice per group). Cytokine statistics represent difference between IFNγ^+^ groups for panel **C**.

In addition to CD4 T cells, the responses in the CD8 T cell compartment were evaluated on Day 49 following incubation with spike-associated peptides overnight via ICS (Figure 5A). Mice vaccinated with the reference vaccine displayed a minor increase in cytokine-producing CD8 T cells (Figure 5B and C), in line with previous literature (Corbett et al., 2020). However, the 10 μg STI-7264 formulation when administered IM led to a robust antigen-specific CD8 T cell response as measured by IFNγ and TNFα. The response was dose-dependent as IM administration of 1 μg STI-7264 led to a minimal CD8 T cell response. Interestingly, when 1 μg STI-7264 was administered via MuVaxx toward draining LNs, the CD8 T cell response was restored to a similar level to that of a 10 μg IM dose (Figure 5B and C). Overall, these results highlight the improved CD8 T cell response observed with this STI-7264 formulation along with the benefit of directing spike-encoding mRNA toward draining LNs to generate CD8 T cell immunity.

### nAbs and memory B cells are generated following STI-7264 vaccination

**Figure 6:**
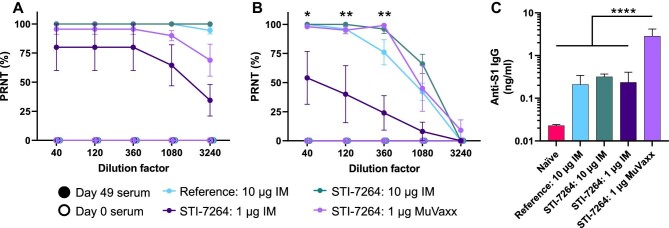
STI-7264 generates circulating nAbs and memory B cells in draining LNs. (**A** and **B**) PRNT dilution curves from mouse serum on Day 49 post prime. (**A**) PRNT curves against the WT virus. (**B**) PRNT curves against the Beta variant. (**C**) Anti-S1 IgG concentrations following *ex vivo* stimulation of draining LNs for 72 h at 15 weeks post booster shot. Data represent one experiment (*n* = 5 mice per group). Statistics in **B** represent the difference between the Day 49 1 μg STI-7264 IM group and all other Day 49 groups.

To assess nAb generation, a plaque reduction neutralization test (PRNT) was performed *in vitro*, where VeroE6 cells were exposed to the live virus in the absence or presence of diluted mouse serum. The PRNT detects plaque formation and is an indication of cell infection by the SARS-CoV-2 virus, whereas the absence of plaque formation represents nAb presence. Each cohort of treatments led to nAb generation by Day 49 against the wild-type (WT) strain (Figure 6A). To investigate protection against the Beta variant, VeroE6 cells were incubated with this strain of the virus. The reference vaccine, 10 μg STI-7264 IM, and 1 μg STI-7264 MuVaxx cohorts, displayed robust protection against this strain up to a 1:360 dilution, whereas the 1 μg STI-7264 IM cohort displayed much lower protection (Figure 6B; Supplementary Figure S2). Taken together, these results show that STI-7264 generates nAbs and lymphatic-mediated delivery via MuVaxx can broaden protection at 1/10 of the IM dose.

To explore vaccine durability, we explored the generation of memory B cells in the lungs and draining LNs 15 weeks after the booster shot. Tissues were collected and single-cell suspensions were stimulated *ex vivo* to promote IgG secretion. Elevated concentrations of anti-S1 IgG were observed in the lungs of the vaccinated mice compared to non-vaccinated mice; however, anti-S1 concentrations were similar regardless of dose or formulation (Supplementary Figure S2). Interestingly, anti-S1 IgG concentrations in draining LNs were substantially elevated in MuVaxx-treated animals (Figure 6C). These data highlight the enhanced generation and/or survival of IgG-secreting B cells in mice where spike-encoding mRNA was delivered more efficiently to draining LNs (i.e. administration with MuVaxx).

## Discussion

Here, we report a novel mRNA-based SARS-CoV-2 vaccine, STI-7264, that induces similar humoral immunity with elevated cellular immunity compared to a reference vaccine formulation when administered IM. Immunity generated with the STI-7264 formulation was dose-dependent as immunity was reduced when the IM dose went from 10 to 1 μg. Interestingly, when administering the same 1 μg STI-7264 formulation via MuVaxx, dose-sparing effects were observed with both humoral and cellular immunity being comparable to a 10 μg IM dose. Similar trends were also observed an order of magnitude lower, highlighting the improved immunogenicity when directing vaccines toward LNs. Previous mRNA-based vaccines have reported dose-dependent side effects with higher doses linked to systemic and local adverse events (Jackson et al., 2020; Mulligan et al., 2020), underscoring an additional advantage of lower dose formulations, i.e. lessening side effects while expanding vaccine access to large populations.

A vaccine that generates durable immunity is another hallmark of an effective vaccine and is a metric we investigated. The serum concentrations of both anti-S1 and anti-RBD IgG waned to a lesser degree in mice treated with MuVaxx relative to those treated with the same IM dose, highlighting improved durability. This is of interest as recent reports have shown declines in SARS-CoV-2 nAbs at 2–3 months after disease onset, as short-lived plasma cells stop producing nAbs (Perreault et al., 2020; Madewell et al., 2022). However, a subset of plasma B cells do differentiate into memory B cells following infection and/or vaccination, leading to persistent germinal center formation within LNs where somatic hypermutation takes place (Lederer et al., 2020; Gaebler et al., 2021; Turner et al., 2021; Laidlaw and Ellebedy, 2022). In line with this, delivery of STI-7264 toward LNs elicited increased nAb concentrations against the Beta variant compared to the dose-matched IM group, suggesting a broadening of the humoral antibody repertoire. Moreover, MuVaxx-mediated delivery of STI-7264 to LNs afforded a 10-fold concentration increase of anti-S1 IgG in stimulated draining LNs at 15 weeks after the booster shot, supporting the notion that efficient delivery of vaccines toward LNs improves memory B cell generation or survival, eliciting an anamnestic response (Moran et al., 2018).

While the most emphasis of the available SARS-CoV-2 vaccines has been focused on the humoral response and nAbs, an ideal vaccine should also generate robust T cell immunity to synergistically protect against infections. In the context of COVID-19, subsets of patients with preexisting SARS-CoV-2-specific T cells have demonstrated rapid viral clearance and less severe disease, highlighting their important role for disease prevention (Sekine et al., 2020; Tan et al., 2021). Additionally, T cell responses against previous betacoronaviruses can persist for decades (Ng et al., 2016; Le Bert et al., 2020) and display cross-reactivity against other betacoronaviruses (Grifoni et al., 2020; Mateus et al., 2020; Weiskopf et al., 2020), underscoring the potential for long-term protection and coverage against variants. In this work, we show that STI-7264 delivered via MuVaxx elicits a strong CD8 T cell response toward SARS-CoV-2 peptides, which may be advantageous for preventing COVID-19 and providing protection against re-infection.

The CD4 Th2 phenotype has been associated with vaccine-associated enhanced respiratory disease (VAERD) in those vaccinated against measles virus and respiratory syncytial virus (Fulginiti et al., 1967; Kim et al., 1969; Munoz et al., 2021). We, therefore, explored the CD4 Th1 vs. Th2 response and IgG2c vs. IgG1 antibody response in vaccinated mice. The response of all vaccinated mice favored a CD4 Th1 response in line with naïve mice. Consistent with the T cell response, the antibody response also skewed toward a Th1 response as measured by the IgG2c:IgG1 ratio in vaccinated mice, with the 10 μg STI-7264 IM and 1 μg STI-7264 MuVaxx cohorts displaying a stronger IgG2c:IgG1 ratio. Mice treated with STI-7264 formulations (high IM dose IM or low MuVaxx dose) may have displayed enhanced anti-viral activity, as mouse IgG2 subclasses have been shown to deliver potent antibody-mediated protection against viruses (Coutelier et al., 1987; Schmitz et al., 2012). Taken together, the Th1 vs. Th2 response shown here suggests a promising activity for avoiding VAERD while promoting anti-viral activity.

Overall, we show preliminary results highlighting improved immunogenicity of a novel mRNA-LNP formulation, STI-7264, with dose-sparing potential enabled when directed toward the draining LN using MuVaxx. This platform has the advantage of not requiring a chemical modification to the drug formulation (e.g. PEGylation, specific buffer, etc.) to be administered (via MuVaxx) or to promote LN accumulation. Thus, MuVaxx may have broad applicability to existing vaccine formulations that are administered via IM injections as well as other drug compounds that are of interest to administer to the skin and/or LNs as demonstrated by using a protein- or mRNA-based vaccine here. Further research is needed to better determine its cellular and humoral durabilities compared to an IM administration along with coverage against known and emerging VoCs. However, the results are promising for continuing forward and evaluating in the clinical setting for vaccinating against SARS-CoV-2.

## Materials and methods

### IVT and purification of RNA

To generate the template for RNA synthesis, the sequence of the SARS-CoV-2 S protein (GenBank: QHD43416.1) was codon-optimized and cloned into the pVAX1-based backbone, which features a 5′-UTR, 3′-UTR, and Poly-A tail. To increase the protein stability, 2P mutations at positions 986–987 were introduced. The plasmid DNA was produced in bacteria, purified, and linearized by a single-site restriction enzyme digestion. The template DNA was purified, spectrophotometrically quantified, and *in vitro* transcribed by T7 RNA polymerase (Cat: M0251, NEB) in the presence of a trinucleotide cap1 analog, m7(3′OMeG) (5′)ppp(5′) (2′OMeA)pG (Cat: N-7113, TriLink), and N1-methylpseudouridine-5′-triphosphate (Cat: N-1081, TriLink) in place of UTP. After the reaction, DNase I (Cat: M0303, NEB) was added to remove the template DNA and the mRNA was purified by LiCl precipitation (Cat: AM9480, ThermoFisher).

### *In vitro* mRNA expression

Monocytes were isolated and differentiated into DCs in the presence of GM-CSF (Cat: 300-03, Peprotech) and IL-4 (Cat: 200-04, Peprotech). Between Day 6 and Day 8, cells were transfected with mRNA by the Neon^TM^ electroporation transfection system (Cat: MPK5000, ThermoFisher). At 24 h post-transfection, the cells were collected and stained with the anti-S antibody STI-2020 in FACS buffer (Dulbecco's phosphate-buffered saline (DPBS) + 0.5% bovine serum albumin) for 30 min on ice. Thereafter, cells were washed twice in FACS buffer and incubated with rat anti-human Fc antibody conjugated to allophycocyanin (Cat: 410712, BioLegend) for 15 min on ice. The cells were washed with FACS buffer and analyzed by the Attune NxT Flow Cytometer (ThermoFisher).

### SARS-CoV-2 virus

SARS-COV-2 viruses were obtained from BEI resources (Washington strain NR-52281; Beta variant NR-54009). VeroE6 monolayers were infected at an MOI of 0.01 in 5 ml of virus infection media (Dulbecco's modified Eagle's medium + 2% fetal calf serum + 1× penicillin/streptomycin). Tissue culture flasks were incubated at 36°C and slowly shaken every 15 min for a 90-min period. Cell growth media (35 ml) was added to each flask and infected cultures were incubated at 36°C/5% CO_2_ for 48 h. The media was then harvested and clarified to remove large cellular debris by room temperature (RT) centrifugation at 3000 rpm.

### Animals and *in vivo* studies

The 6- to 12-week-old C57Bl6 mice were purchased from the Jackson Laboratory. All protocols were approved by the Institutional Animal Care and Use Committee (IACUC). Mice were injected with the indicated administration technique under isoflurane anesthesia in the right hind flank area for IM injections and in the right dorsal area for MuVaxx injections. For imaging studies, ICG (Sigma) was dissolved at 2.5 mg/ml in deionized water and injected using MuVaxx with a NIRF camera used to collect images 5 min following injection. For OVA vaccine studies, 10 μg OVA (Cat: VAC-POVA, InvivoGen) and 8 μg CpG (Cat: TLRL-1826-1, InvivoGen) were administered to mice on Days 0 and 14. Peripheral blood was collected from anesthetized mice once a week via the submandibular route. Reference mRNA-LNP vaccine is the same construct as a EUA-cleared compound.

### ELISA assays

To assess spike-specific antibodies, S1 (Cat: 40591-V08H, Sino Biological) or RBD (Cat: 40592-V08B, Sino Biological) protein was coated on half-area high binding plates (Cat: N503, Thermo) at 1 μg/ml overnight at 4°C. Plates were washed three times with ELISA wash buffer (Thermo), pre-blocked with casein blocker (Cat: 37528, Thermo) for 1 h at RT, and washed one time with ELISA wash buffer. Mouse sera were diluted in casein blocker and transferred to ELISA plates for 1 h at RT followed by three wash steps. Secondary antibodies of horse radish peroxidase (HRP)-conjugated rabbit anti-mouse IgM (μ chain), HRP-conjugated rabbit anti-mouse IgG (Fcγ), HRP-conjugated goat anti-mouse IgG1, or HRP-conjugated goat anti-mouse IgG2c were added to ELISA plates for 1 h at RT followed by six wash steps. Plates were developed with TMB substrate solution (Cat: 34021, Thermo) for ∼10 min at RT and stopped with 2 M sulfuric acid. The absorbance was measured at 450 nm using a BioTek Cytaktion 5 plate reader. For IgG (Fcγ), a standard curve was generated using anti-RBD PAb (Cat: 40592-MP01, Sino Biological) or anti-S1 (Cat: MAB105405, R&D Systems) starting at 3000 ng/ml with 3-fold serial dilutions. For IgM (μ chain), IgG1, and IgG2c, serial fold dilutions were run and titers were determined using an absorbance cutoff of 0.7 OD.

### PRNT

Simian VeroE6 cells were plated at 18 × 10^3^ cells/well in a flat-bottom 96-well plate at a volume of 200 μl/well. After 24 h, a serial dilution of seropositive blood serum was prepared in 100 μl/well at twice the final concentration desired, and the live virus was added at 1000 PFU/100 μl of SARS-CoV-2 and subsequently incubated for 1 h at 37°C in a total volume of 200 μl/well. Cell culture media was removed from cells and serum/virus premix was added to VeroE6 cells at 100 μl/well and incubated for 1 h at 37°C. After incubation, 100 μl of ‘overlay’ (1:1 of 2% methylcellulose (Sigma) and culture media) was added to each well and incubation commenced for 3 days at 37°C. Plaque staining using crystal violet (Sigma) was performed following 30 min of fixing the cells with 4% paraformaldehyde (Sigma) diluted in PBS. Plaques were assessed using a light microscope (Keyence).

### Peripheral blood T cell ICS

ICS was performed at the indicated time points following the booster shots for IFNγ, TNFα, and IL-4. Whole blood was stimulated for 6 h with 1 μg/ml of SIINFEKL (Sigma) or overnight with 1 μg/peptide per well of spike-associated peptides (Cat: 130-127-951, Miltenyi Biotec Peptivator) at 37°C, 5% CO_2_ in the presence of brefeldin A (Biolegend) and monensin (Biolegend). Following stimulation, whole blood was incubated with red lysis buffer (Cat: A10492-01, Gibco) at RT. Cells were permeabilized using Intracellular Staining Perm Wash Buffer (Cat: 421002, Biolegend). Cells were stained with phycoethyrin (PE) anti-mouse IFNγ (Cat: 505808, Biolegend), fluorescein isothiocyanate anti-mouse TNFα (Cat: 506304, Biolegend), BV421 anti-mouse IL-4 (Cat: 504127, Biolegend), allophycocyanin-Cy7 anti-mouse CD3 (Cat: 100222, Biolegend), PE-Cy7 anti-mouse CD4 (Cat: 25-0041-82, Invitrogen), and allophycocyanin anti-mouse CD8a (Cat: 100712, Biolegend). Naïve mice (non-vaccinated mice) were used as negative controls. Cells were then run on a Beckman Coulter CytoFLEX instrument and analyzed via FlowJo V10 software.

### Long-term immunity experiments

Fifteen weeks after the booster shot, mice were sacrificed, and draining LNs were harvested for single-cell suspension generation. Draining LNs were generated by mechanically disrupting LNs through a 70-μm nylon cell strainer. Cells were then washed three times, each with 20 ml of cold DPBS, to ensure the removal of any IgG present in the draining LNs. After wash steps, lymphocytes were stimulated for 72 h in 1 ml of complete media (RPMI 1640 + 10% fetal bovine serum + 1% penicillin/streptomycin) with the addition of Resiquimod (R848) (1 μg/ml) and IL-2 (10 ng/ml) in 24-well plates. After 72 h, plates were spun down and supernatants were collected for ELISA measurement as discussed above.

### Statistics

Statistical significance of differences between experimental groups was determined with Prism software (Graphpad). All data are expressed as mean ± standard error mean (SEM). ****P < 0.0001, ***P < 0.001, **P < 0.01, and *P < 0.05 by unpaired two-tailed *t*-tests or one- or two-way analysis of variance.

## Compliance with ethics guidelines

All animal experiments were performed in accordance with IACUC guidelines and approvals.

## Data availability

The datasets used and/or analyzed during the current study are available from the corresponding author on reasonable request.

## Supplementary Material

mjac041_Supplemental_File

## Notes

**Author contributions:** D.M.F., R.C., N.S., and H.X. conceptualized and designed experiments. D.M.F., S.K., M.S., Q.H., X.L., R.C., W.L., H.W., H.S., N.S. H.X. performed experiments and acquired the data. D.M.F., R.C., H.X., Q.H., X.L., B.C., R.R., and H.J. analyzed and interpreted the data. D.M.F., R.C., and N.S. wrote the paper. All named authors meet the International Committee of Medical Journal Editors (ICMJE) criteria for authorship for this study, take responsibility for the integrity of the work as a whole, and have given their approval for this version to be published.
