# Supplementary material for: Delivering an mRNA vaccine using a lymphatic drug delivery device improves humoral and cellular immunity against SARS-CoV-2
Source: J Mol Cell Biol. 2022 Jul 8;14(6):mjac041. doi: 10.1093/jmcb/mjac041 (PMC9753907; doi:10.1093/jmcb/mjac041)
Supplement: mjac041_Supplemental_File [file mjac041_supplemental_file.pdf]

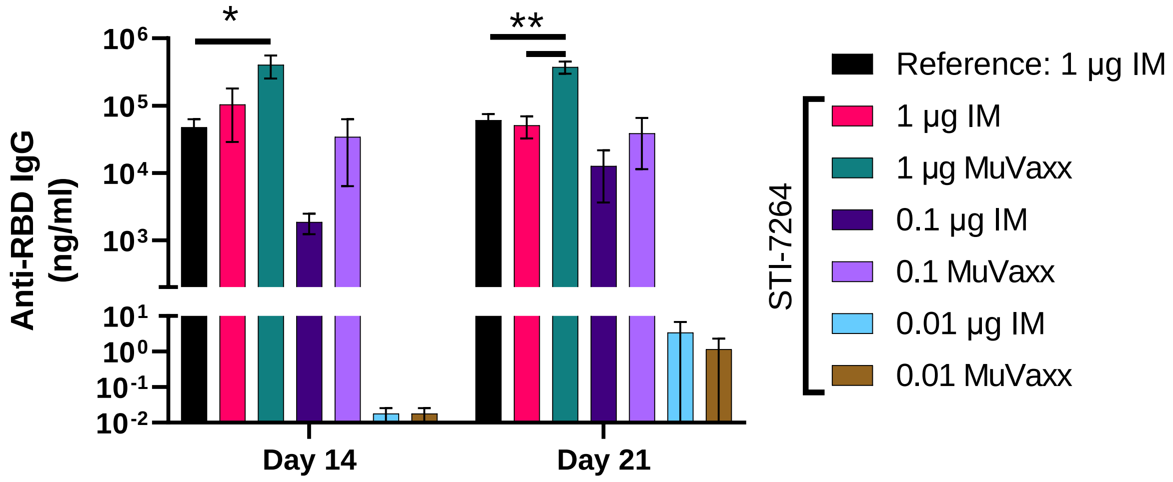

Supplementary Figure S1 MuVaxx enables dose sparing of STI-7264 vaccine formulation. Mice were injected with indicated dose and formulation using an IM or MuVaxx administration. Serum was then collected on day 14 and 21 post prime dose and anti-RBD IgG was measured. Data represents one experiment (*n*=4–5 mice per group).

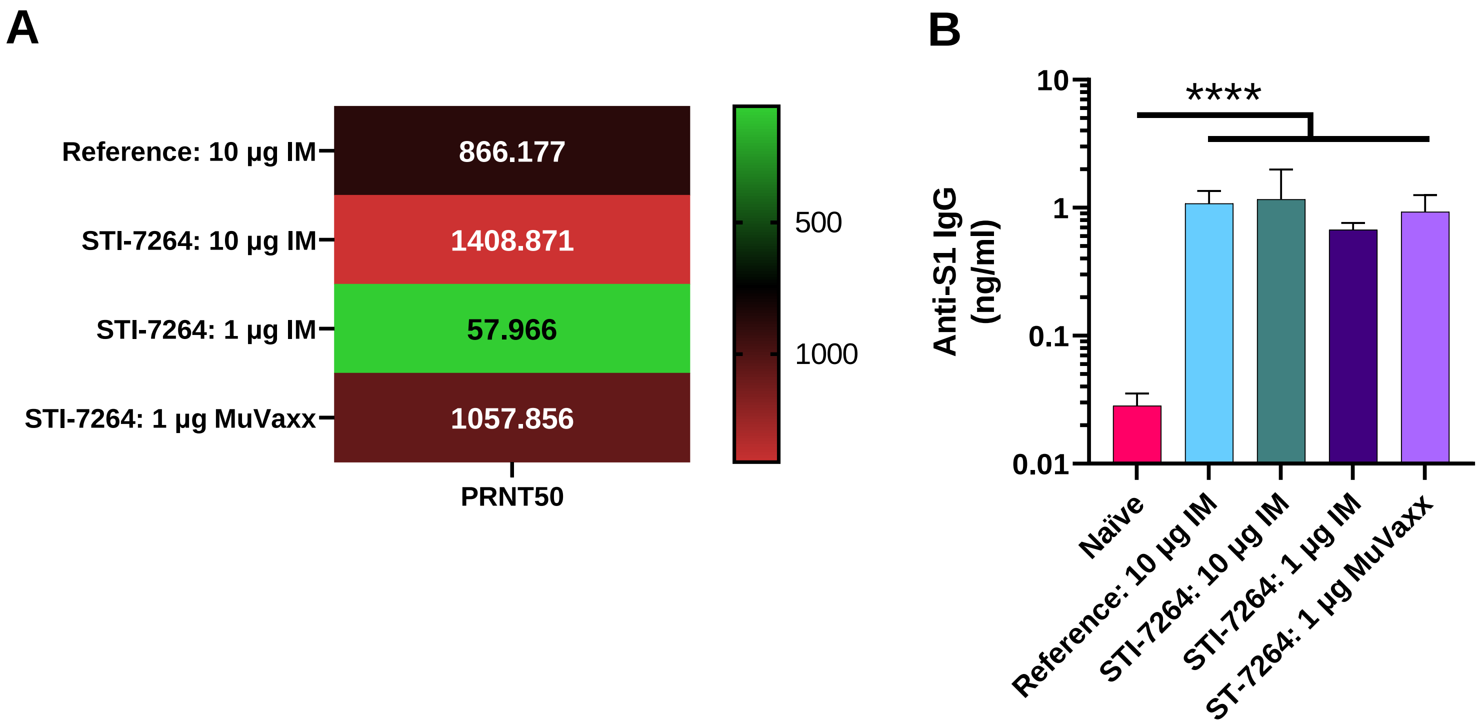

Supplementary Figure S2 PRNT50 values and anti-S1 IgG produced by memory B cells in lungs following vaccination. (**A**) Quantified PRNT50 values from Figure 6B. (**B**) Anti-S1 IgG concentrations following *ex vivo* stimulation of lungs for 72 hours after 15 weeks post booster shot. Data represents one experiment (*n*=5 mice per group).
